# Supplementary material for: Closing Tobacco Treatment Gaps for Rural Populations: The Role of Clinic-Based Pharmacists at a Federally Qualified Health Center
Source: Pharmacy (Basel). 2025 Jan 26;13(1):10. doi: 10.3390/pharmacy13010010 (PMC11858977; doi:10.3390/pharmacy13010010)
Supplement: Supplementary file 1 [file pharmacy-13-00010-s001.zip › pharmacy-3393185-supplementary.pdf]

Supplementary Materials

Survey - English Version

Smoking and Tobacco Use – Patient Survey S1

Instructions

Answer questions as they relate to you. For most answers, check the box(es) most applicable to you or fill in the blanks.

Section A: Smoking Habits/ Desire to Quit

1. Do you NOW smoke cigarettes, vape products or use any other forms of tobacco: (Select only one.)

- ☐ Everyday
- ☐ Somedays
- ☐ Not at all

2. Which of the following forms of nicotine do you use daily or some days? (Select all that apply)

- ☐ Cigarettes
- ☐ Smokeless tobacco
- ☐ Vaping
- ☐ Others: (please specify): ___

3. On average, how many cigarettes do you smoke per day?

____

4. For how many years have you smoked, vaped, or used tobacco?

- ☐ Less than one year
- ☐ 1 to 5 years
- ☐ 6 to 10 years
- ☐ 11-20 years
- ☐ More than 20 years
- ☐ Unsure

5. Do you currently live with someone who smokes?

- ☐ Yes
- ☐ No

6. Do you ever smoke inside your home (house, apartment)?

- ☐ Yes
- ☐ No

7. During your lifetime, have you ever tried to quit smoking?

- ☐ Yes
- ☐ No

If you answered "NO" to question 7, please SKIP questions 8,9 & 10

8. When was your last attempt to quit smoking, vaping, or other tobacco products?

- ☐ More than 12 months ago
- ☐ Less than 12 months ago

9. During your lifetime, have you ever used a medicine to help you quit smoking?

- ☐ Yes
- ☐ No
- ☐ Not sure

10. Please select all that apply. The following are smoking replacements that I have tried in the past:

- ☐ Nicotine gum
- ☐ Nicotine lozenge
- ☐ Nicotine transdermal patch
- ☐ Nicotine nasal spray
- ☐ Nicotine oral inhaler
- ☐ Bupropion SR (Zyban)
- ☐ Varenicline (Chantix)
- ☐ None

11. Are you considering quitting?

- ☐ Yes, in the next month
- ☐ Yes, in the next 6 months
- ☐ Yes, but not in the next 6 months
- ☐ No, I am not quite ready

12. What do you find most challenging about quitting smoking? (Select all that apply)

- ☐ I am worried about weight gain
- ☐ I am worried about withdrawal symptoms
- ☐ I cannot afford medications to help me quit
- ☐ My peers/ family members smoke around me
- ☐ Others (please specify): ____

Section B: Counseling Received from Clinic Staff Member

1. I see my primary care provider

- ☐ Less than once yearly
- ☐ Once a year
- ☐ Twice a year
- ☐ Three times a year
- ☐ More than three times a year

2. Has your primary care provider asked you if you smoke, vape, or use other types of tobacco?

- ☐ Yes
- ☐ No

3. On average, how much time does your primary care provider spend talking with you about tobacco use?

- ☐ No time
- ☐ Up to 3 minutes
- ☐ 3-5 minutes
- ☐ >6 minutes

4. I am aware that I can make an appointment with my provider to specifically discuss ways to stop smoking

- ☐ Yes
- ☐ No

5. Other than my primary care provider in the clinic, I have been advised to quit smoking by: (Select all that apply)

- ☐ Nurses
- ☐ Pharmacist
- ☐ None
- ☐ Others (please specify): --

6. Has a pharmacist or pharmacy staff ever asked you about your smoking habits?

- ☐ Yes
- ☐ No

7. I am aware that pharmacists can assist me to stop smoking

- ☐ Yes
- ☐ No

8. I would be interested in establishing care with a pharmacist to help me stop smoking.

- ☐ Yes
- ☐ No

If you answered "No" to question 8, please SKIP question 9

9. It would be more convenient to establish care with your community pharmacist to quit smoking.

- ☐ Agree
- ☐ Disagree
- ☐ Unsure

10. It would be more convenient to have my appointments held online via telehealth to discuss my smoking habits?

- ☐ Yes
- ☐ No
- ☐ No preference

11. I intend to ask my pharmacist for help with regards to stopping smoking.

- ☐ Yes
- ☐ No

12. I intend to ask my pharmacist about the benefits and risks of smoking cessation medications to assist in my quit attempt.

- ☐ Yes
- ☐ No
- ☐ Unsure

13. I would be interested in group sessions/ discussions with the pharmacist and others in the community who smoke to motivate me to quit smoking.

- ☐ Yes
- ☐ No
- ☐ Unsure

14. People who are important to me would be supportive of me receiving aid from a pharmacist to quit smoking.

- ☐ Agree
- ☐ Disagree
- ☐ Unsure

Section C: Demographics

1. Which of the following describes you?

- ☐ Male
- ☐ Female
- ☐ Non-binary / third gender
- ☐ Prefer not to respond

2. What is your age?

- ☐ 18-24
- ☐ 25-34
- ☐ 35-44
- ☐ 45-54
- ☐ 55-64
- ☐ ≥65
- ☐ Prefer not to respond

3. Which of the following best describes you (select all that apply)?

- ☐ White/ Caucasian
- ☐ Asian
- ☐ Black/ African American
- ☐ Native Hawaiian or other Pacific Islander
- ☐ American Indian/ Alaska native
- ☐ Others
- ☐ Prefer not to respond

4. What language do you feel most comfortable using when speaking to your healthcare team?

- ☐ English
- ☐ Spanish

☐ Others (please specify):____

5. Would you be interested in being contacted by a member of UW Family Medicine Residency Clinic's Pharmacy staff to receive more information about quitting?

☐ Yes. If yes, please provide your name and contact information in the contact sheet provided (separate from the survey to ensure anonymity)
☐ No

Thank you so much for taking the time to complete our survey! We hope you have a wonderful day!

Survey - Spanish Version

Encuesta acerca del consumo de Tabaco

Instrucciones

Responda a las siguientes preguntas de acuerdo a su experiencia. En la mayoría de las respuestas, marque la(s) casilla(s) más apropiadas o llene el espacio en blanco.

Sección A: Hábitos de Fumar/ Deseo de dejar de fumar

1. En este momento de su vida, ¿Usted fuma cigarrillos, vapea o utiliza otros productos de tabaco? (Marque solo una casilla.)

- ☐ Todos los días
- ☐ Algunos días
- ☐ Nunca

2. ¿Cuál de los siguientes productos de nicotina consume usted diariamente o algunos días? (Seleccione todas las opciones que correspondan.)

- ☐ Cigarrillos
- ☐ Tabaco sin humo (de mascar o rapé)
- ☐ Vape
- ☐ Otro: (Por favor, especifique): ___

3. ¿En general, cuantos cigarrillos fuma usted por día?

____

4. ¿Por cuántos años usted ha fumado, usado vape u otros productos de tabaco?

- ☐ Menos de un año
- ☐ 1 a 5 años
- ☐ 6 a 10 años
- ☐ 11 a 20 años
- ☐ Más que 20 años
- ☐ No estoy seguro

5. ¿Usted vive actualmente con alguien que fuma?

- ☐ Sí
- ☐ No

6. ¿Usted fuma adentro de su hogar (casa, apartamento)?

- ☐ Sí
- ☐ No

7. Durante su vida, ¿En algún momento ha intentado dejar de fumar?

- ☐ Sí
- ☐ No

Si respondió “NO” a la pregunta 7, por favor no conteste las preguntas 8, 9 y 10. Continúe en la pregunta 11.

8. ¿Cuándo fue su último intento de dejar de fumar, utilizar vape u otros productos de tabaco?

- ☐ Hace más de 12 meses
- ☐ Hace menos de 12 meses

9. ¿En su vida, alguna vez ha utilizado un medicamento para ayudarle a dejar de fumar?

- ☐ Sí
- ☐ No
- ☐ No estoy seguro

10. Por favor seleccione todas las opciones que correspondan. En el pasado he usado estos sustitutos en ocasiones cuando traté de dejar de fumar:

- ☐ Chicle de nicotina
- ☐ Pastilla/rombo de nicotina
- ☐ Parche transdérmico de nicotina
- ☐ Aerosol nasal de nicotina
- ☐ Inhalador oral de nicotina
- ☐ Bupropion SR (Zyban)
- ☐ Varenicline (Chantix)
- ☐ Ninguno

11. ¿Está considerando dejar de fumar?

- ☐ Sí, en el próximo mes
- ☐ Sí, en los próximos 6 meses
- ☐ Sí, pero no en los próximos 6 meses
- ☐ No, no estoy listo todavía

12. ¿Qué le preocupa más acerca de dejar de fumar? (Seleccione todas las opciones que correspondan.)

- ☐ Me preocupa el aumento de peso
- ☐ Me preocupan los síntomas de abstinencia
- ☐ No puedo pagar los medicamentos que me pueden ayudar a dejar de fumar
- ☐ Mis compañeros/familiares fuman a mi alrededor
- ☐ Otros (especifíquese): ____

Sección B: Consejo recibido de un empleado de la Clínica

1. Yo veo a mi médico de cabecera:

- ☐ Menos de una vez al año
- ☐ Una vez al año
- ☐ Dos veces al año
- ☐ Tres veces al año
- ☐ Más de tres veces al año

2. ¿Su médico de cabecera le ha preguntado si fuma, vapea o usa otros tipos de tabaco?

- ☐ Sí
- ☐ No

3. ¿En general, cuánto tiempo pasa su médico de cabecera hablando sobre su uso de tabaco?

- ☐ Nada de tiempo
- ☐ Hasta 3 minutos
- ☐ De 3 a 5 minutos
- ☐ Más de 6 minutos

4. Yo sé que puedo hacer una cita con mi médico de cabecera para discutir específicamente métodos para dejar de fumar.

- ☐ Sí
- ☐ No

5. Aparte de mi médico de cabecera, estos otros empleados de la clínica me han aconsejado dejar de fumar: (Seleccione todas las opciones que correspondan.)

- ☐ Enfermeras
- ☐ Farmacéutico
- ☐ Ninguno
- ☐ Otros (especifique): ____

6. ¿Alguna vez un farmacéutico o personal de farmacia le ha preguntado sobre sus hábitos de fumar?

- ☐ Sí
- ☐ No

7. Yo sé que los farmacéuticos me pueden asistir a dejar de fumar.

- ☐ Sí
- ☐ No

8. Estaría interesado en comenzar un tratamiento en la clínica con un farmacéutico para que me ayude a dejar de fumar.

- ☐ Sí
- ☐ No

Si respondió "No" a la pregunta 8, por favor salte la pregunta 9 y continúe en la pregunta 10.

9. Sería más conveniente comenzar un tratamiento con un farmacéutico de la comunidad para que me ayude a dejar de fumar.

- ☐ De acuerdo
- ☐ Desacuerdo
- ☐ No estoy seguro

10. Sería más conveniente tener mis citas por internet a través de Telesalud para discutir mis hábitos de fumar.

- ☐ Sí
- ☐ No
- ☐ No tengo preferencia

11. Tengo la intención de pedirle ayuda a mi farmacéutico para dejar de fumar.

- ☐ Sí
- ☐ No

12. Tengo la intención de preguntarle a mi farmacéutico acerca de los beneficios y riesgos de los medicamentos disponibles para ayudarme a dejar de fumar.

- ☐ Sí
- ☐ No
- ☐ No estoy seguro

13. Estaría interesado en sesiones de grupo/ discusiones con el farmacéutico y otras personas que fuman en la comunidad para que me motive a dejar de fumar.

- ☐ Sí
- ☐ No
- ☐ No estoy seguro

14. Las personas que son importantes para mí me apoyarían a recibir ayuda de un farmacéutico para dejar de fumar.

- ☐ De acuerdo
- ☐ Desacuerdo
- ☐ No estoy seguro

Sección C: Demografía

1. ¿Cuál de las siguientes opciones lo describen?

- ☐ Masculino
- ☐ Femenino
- ☐ No binario / Tercer género
- ☐ Prefiero no responder

2. ¿Cuántos años tiene?

- ☐ 18-24
- ☐ 25-34
- ☐ 35-44
- ☐ 45-54
- ☐ 55-64
- ☐ más de 65
- ☐ Prefiero no responder

3. ¿Cuál de las siguientes opciones le describe (seleccione todas las que correspondan)?

- ☐ Blanco / Caucásico
- ☐ Asiático
- ☐ Negro / Afroamericano
- ☐ Hawaiano nativo u otros isleños del Pacifico
- ☐ Amerindio / Nativo de Alaska
- ☐ Otros
- ☐ Prefiero no responder

4. ¿En qué lenguaje se siente más cómodo cuando habla con su equipo médico?

- ☐ Inglés
- ☐ Español
- ☐ Otro (especifique): ____

5. ¿Le interesaría ser contactado por un miembro farmacéutico de la UW Clínica de Residentes de Medicina Familiar para recibir más información sobre dejar de fumar?

- ☐ Sí.

[Por favor escriba su nombre e información de contacto en la página separada de la encuesta para garantizar anonimato.]

- ☐ No

¡Muchas gracias por tomar el tiempo para completar esta encuesta! ¡Esperamos que tenga un día maravilloso!
